# Understanding components of mobility during the COVID-19 pandemic

**DOI:** 10.1098/rsta.2021.0118

**Published:** 2022-01-10

**Authors:** Peter Edsberg Møllgaard, Sune Lehmann, Laura Alessandretti

**Affiliations:** ^1^ Department of Applied Mathematics and Computer Science, Technical University of Denmark, Kongens Lyngby, Denmark; ^2^ The Center for Social Data Science, University of Copenhagen, Copenhagen, Denmark; ^3^ Statistics Denmark, Copenhagen, Denmark

**Keywords:** human mobility, COVID-19, non-negative matrix factorization

## Abstract

Travel restrictions have proven to be an effective strategy to control the spread of the COVID-19 epidemics, in part because they help delay disease propagation across territories. The question, however, as to how different types of travel behaviour, from commuting to holiday-related travel, contribute to the spread of infectious diseases remains open. Here, we address this issue by using factorization techniques to decompose the temporal network describing mobility flows throughout 2020 into interpretable components. Our results are based on two mobility datasets: the first is gathered from Danish mobile network operators; the second originates from the Facebook Data-For-Good project. We find that mobility patterns can be described as the aggregation of three mobility network components roughly corresponding to travel during workdays, weekends and holidays, respectively. We show that, across datasets, in periods of strict travel restrictions the component corresponding to workday travel decreases dramatically. Instead, the weekend component, increases. Finally, we study how each type of mobility (workday, weekend and holiday) contributes to epidemics spreading, by measuring how the effective distance, which quantifies how quickly a disease can travel between any two municipalities, changes across network components.

This article is part of the theme issue ‘Data science approaches to infectious disease surveillance’.

## 1. Introduction

Throughout the COVID-19 pandemic, governments worldwide have restricted individual mobility for their citizens with the goal of reducing social contacts and thus limit spread of the virus. Some of these interventions, e.g. travel bans within and across national borders and home-isolation orders, target travel behaviour specifically. Others, such as the closure of schools and businesses, the cancellations of events and restrictions on gatherings, result in a reduction of travel as an indirect consequence.

Analyses of call detail records and GPS trajectories extracted from mobile phones are an effective way to monitor and model the effect of population-scale interventions on behaviour and on the spread of the epidemics [1]. Studies based on phone data have shown that travel restrictions can strongly limit (or fully contain) the spread of diseases [2–9], even though restrictions have not proven equally effective everywhere. In low-income areas, for example, people were not able to reduce their mobility as sharply [6,7,9,10], and tended to visit more crowded points of interest [5]. Some of the mechanisms responsible for this observed disparity may be rooted constraints affecting the ability to respond (e.g. capacity to work from home, take paid or unpaid time off of work, and draw on savings to limit shopping trips to meet basic needs). It has further been shown [9,11] that travel restrictions had stronger impact on long-range distance trips [11]. However, the effect of restrictions on mobility behaviour is not fully understood, partly because, while travelling serves different purposes, from daily commuting and errands to going on holiday, it is unclear how restrictions have affected these various components of travel behaviour. Studies based on aggregated mobile phone data have focused on general mobility trends, and overall network effects [11], disregarding the role played by different aspects of human travel. On the other hand, studies based on high resolution position cover individual cities or regions, and thus describe only commuting and day-to-day mobility, or do not focus on the connectivity between regions.

In this work, we study how non-pharmaceutical interventions have affected different components of travel behaviour using two large-scale datasets collected from mobile phones. The first dataset consists of the daily number of trips within and between Danish municipalities throughout 2020, estimated using data collected by Danish mobile network operators (see Material and Methods) [12]. The second dataset consists of the number of trips within and between provinces in France, Italy and Spain estimated using data collected by Facebook [13]. We propose a new way to identify structural components in human mobility using factorization techniques. We show that, across different datasets, mobility patterns are well described as the combination of three components: the first mainly capturing commuting and work-day travel, the second describing weekend trips, and the latter describing holiday. We show how these different components contributed to the *effective distance*, which determines how long it takes for a disease to diffuse across time and space.

### (a) Timeline of non-pharmaceutical interventions in Denmark

Travel patterns in Denmark were strongly affected by mobility restrictions throughout the COVID-19 pandemic. Denmark experienced two epidemic waves which prompted the government to introduce strict non-pharmaceutical interventions across the country: the first between February and May 2020, and the second between September 2020 and February 2021. In this work, we focus on travel patterns in the period between February and December 2020, thus our focus will mostly be on the first wave. Events unfolded as follows [14,15]. Not long after the discovery of the first COVID-19 local case on 27 February 2020, Denmark was the first country to impose country-wide non-pharmaceutical interventions. Starting from 16 March, interventions included the following: closure of borders, closure of schools, universities and cultural institutions, bans on social gatherings, closure of non-essential retail businesses, bars and restaurants. Between 13 April and 27 May 2020, restrictions were gradually released. Throughout the summer, with few exceptions, such as discotheques and nightclubs remaining closed, activities in Denmark returned closer to normal. In September, the surge of new cases led the government to introduce new nationwide restrictions, substantially milder than those introduced in the spring, including travel bans to foreign countries, work-from-home orders for public sector employees, the closure of bars and restaurants at midnight, the requirement to wear face masks and bans on social gatherings. In November, stricter local restrictions were imposed in the region of Northern Jutland due to the concerns created by the surge of the mink variant of the virus. Starting from mid-December, with the infections on the rise across the country, the government re-introduced stricter nationwide restrictions, including the closure of non-essential businesses and schools. In the following, we refer to periods of stringent travel restrictions as periods of ‘lockdown’. We identified periods of ‘lockdown’ using the stringency data released by the Oxford COVID-19 Government Response Tracker (for further details see electronic supplementary material, figure S6 and table S2) [16].

## 2. Results

### (a) The components of human mobility

**Figure 1.**
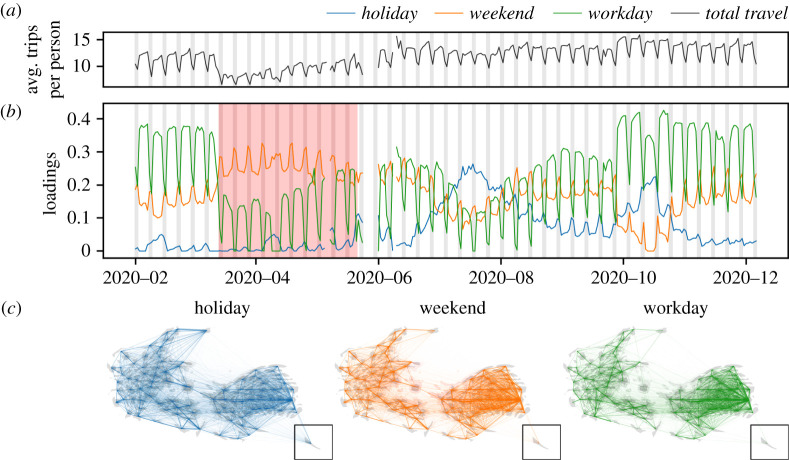
Mobility patterns in Denmark can be decomposed into three interpretable components. (*a*) Estimated number of trips per person over time. (*b*) The loadings ${\text{H}}_{k}$ of the three components of mobility identified by NMF over time: the *holiday* component (blue, for k=1), the *weekend* component (orange, for k=2) and the *workday* component (green, for k=3). The red area indicates the period of ‘lockdown’ (see electronic supplementary material, Supplementary Information figure S6). (*c*) The memberships ${\text{W}}_{k}^{⊺}$ of links representing the number of trips between cities to the three components *holiday*, *weekend* and *workday* (from left to right). Links are represented on cartograms [17] displaying the map of Denmark (see Material and methods). For visualization purposes, the link widths are proportional to the $x^{1.5}$, where x is the membership to the component. (Online version in colour.)

Travel in Denmark was substantially affected by non-pharmaceutical interventions, with the number of total trips decreasing by 27% following the introduction of the first ‘lockdown’ in mid-March 2020, and then a gradual increase as the ‘lockdown’ was released between mid-April and mid-May (see figure 1*a*, black line). While mobility encompasses a wide range of behaviours, from travelling on holiday to commuting [18], the effect of restrictions on these different behaviours has not been systematically investigated. The visual inspection of the number of trips over time (figure 1*a*) reveals that the total amount of travel was subject to dramatic changes throughout 2020, but does not allow to understand further the impact of restrictions on travel. In this section, focusing on travel patterns in Denmark, we use non-negative matrix factorization (NMF) as a systematic way to investigate how different components of mobility have been affected by travel restrictions.

Our dataset describes the total number of daily trips between any pair of Danish municipalities over time (see also Data description). We summarize the data in the *mobility matrix* A, with dimensions $N^{2}\times D$, where N=98 is the number of municipalities in Denmark and D=300 is the total number of days considered. Each entry $A_{i,t}$ of the matrix corresponds to the normalized number of trips between a pair of municipalities i on day t (see Material and methods for details on the normalization). A pair of municipalities is defined as i=(o,d), where o corresponds to the origin and d to the destination of the trip. Note that there are $N^{2}$ pairs, because, in general, the number of trips between o and d on a given day differs from the number of trips between d and o on the same day. When o=d, the data correspond to the number of trips within the same municipality. Each row ${\text{A}}_{i}$ of the matrix is a vector of D elements, containing the number of trips over time for a pair i=(o,d) of municipalities. Each column ${\text{A}}_{t}^{⊺}$ is a vector containing the number of trips between any pair of municipalities in a particular day t, where ${\text{A}}^{⊺}$ denotes the transpose of matrix A.

A natural way to identify components in travel behaviour is to use NMF, a widely used technique for identifying interpretable structures in non-negative data [19]. Formally, we achieve the decomposition by approximating the mobility matrix A as the product of two non-negative matrices H and W, such that A≈WH, where the dimension K (i.e. the number of columns) of the factorization matrices H and W is smaller than the dimensions of the data matrix (see Material and methods for further details). This implies that we can express each term of the mobility matrix as

$$A_{i,t}\approx \sum_{k=0}^{K}W_{i,k}H_{k,t},$$

where $W_{ik}$ quantifies the membership of a given pair of municipalities i to a given component k and $H_{kt}$ quantifies the activity level of each component at time t. Hence, in the following, we refer to the matrix W as the matrix of *memberships*, and to the matrix H as the matrix of *loadings*. This interpretation of the two matrices comes directly from the factorization, due to the dimensions of the W and H matrices, because W has dimensions $N^{2}\times K$ and H has dimensions K×D. Furthermore, understanding the matrix W as the matrix of memberships is standard in the literature on non-negative matrix factorization applied to networks [20,21]. In order to find the matrices H and W, we performed matrix factorization, thus numerically minimizing the distance

$$||\text{A}-\text{WH}|{|}_{F}^{2},$$

where $||\text{X}|{|}_{F}$ is the Frobenius norm of matrix X (see Material and methods for further details). Note that NMF was used successfully to detect structural components in a wide range of objects of interest, including networks [22], images [19] and textual data [23].

We apply the NMF decomposition described above, setting K=3 using a method based on cross-validation [24] (see Material and methods and electronic supplementary material, figure S3). Our hypothesis is that the structural components of mobility identified by NMF can be interpreted [19] as corresponding to different types of mobility behaviours, such as commuting, travelling for leisure and trips related to going on holiday. We test this hypothesis by quantifying how active the different components are during specific periods: namely weekends, workdays, holidays, and epidemic waves. Formally, we introduce four vectors. The first three are binary vectors and represent *workday*, *weekend* and *holiday*, where each element correspond to a day. The non-zero elements in each of the three vectors respectively correspond to workdays, weekends, and holidays (see electronic supplementary material for the full list of holidays table S1). Finally, in order to study how the components of mobility relate to the unfolding of the epidemics, we introduce the *covid-19 cases* vector, containing the daily deaths numbers by COVID-19 in Denmark shifted by 13 days [25] to represent the proportion of infected people at any given day (the COVID-19 cases dataset is downloaded from the website Our World in Data [26] using the Python API [27]). Here, we use the number of deaths over time rather than infections, as the latter is strongly affected by the testing capacity, and we apply the square root to the *covid-19 cases* vector, because the same transformation is applied to the mobility data (see Material and methods). Finally, for each component of mobility detected by NMF, i.e. for each value of k∈{1,2,3}, we compute the correlation between the loadings over time, ${\text{H}}_{k}$, and each of the four vectors defined above. Note that, when computing the correlation between the components and the *weekend* and *workday* vectors, we first de-trend the components by subtracting a moving average over a window of seven days, since we are interested in understanding weekly patterns. Furthermore, we remark that the order of the three components is arbitrary and does not carry any meaning.

Interestingly, we find that the NMF components are highly correlated with the vectors defined above, confirming the hypothesis that the NMF identifies structures that relate to different types of mobility behaviour (see also electronic supplementary material, figure S4). The first component, for k=1, correlates positively with the *holiday* and *weekend* vectors, and negatively with the *workday* and *COVID-19 cases* vectors suggesting it captures leisure trips. The second component, for k=2, correlates positively with the *weekend* and the *covid-19 cases* vectors, and correlates negatively with the others. We note that the correlation with the *weekend* vector is much higher compared with the correlation with the *covid-19 cases* vector. The third component, for k=3, correlates positively with the *workdays* vector, and negatively with the other vectors, implying it captures commuting trips (table 1). In light of these observations, it is reasonable to conclude that each NMF component roughly describes an aspect of mobility behaviour. Thus, we name each component after the vector with the highest correlation: we refer to the first component as the *holiday* component, to the second component as the *weekend* component and to the third component as the *workday* component. Interestingly, the correlation analysis for the *covid-19 vector* (table 1) suggests that, in periods of high epidemic activity, holiday and workday travel were reduced while weekend travel patterns increased. As expected, the total number of trips over time (see last line in table 1 and figure 1*a*) correlates positively with the *workday* vector, and negatively with the *weekend* and *covid-19 cases* vectors.

**Table 1.** Pearson correlation between each NMF component (for k∈{1,2,3}) and the total trips (see table rows), with the *weekend*, *workday*, *holiday* and *covid-19 cases* vectors (see table columns). Unless stated in the table as non-significant (ns), correlations are significant at α=0.05.

	weekends	workdays	covid-19 cases	holiday
k=1	0.52	−0.52	−0.38	*0.64*
k=2	*0.87*	−0.87	0.32	−0.19
k=3	−0.92	*0.92*	−0.09 (ns)	−0.37
*total trips*	−0.49	0.49	−0.42	−0.04 (ns)

In figure 1*b*, we show the evolution of the three components ${\text{H}}_{k}$ over time. The findings presented in table 1 can be clearly observed in the figure, with the *weekend* and *workday* components following weekly patterns, and the *holiday* component following the Danish holiday calendar. We observe that, during the ‘lockdown’ period, the loading of the *weekend* component increases, moving from 0.15, on average, before lockdown to 0.26, on average, during lockdown, implying an increase of 69%. The workday component, instead, decreased from 0.31 before lockdown, to 0.11 during the lockdown, implying a decrease of 64%.

The decomposition obtained via NMF can be naturally used to define three networks, where nodes are municipalities and weighted links connect origin-destination pairs, with weights corresponding to the memberships to the *weekend*, *workday* and *holiday* component, respectively. More formally, for each component k, the vector ${\text{W}}_{k}^{⊺}$, corresponds to the weighted adjacency list [28] defining the weighted network. We visualize the networks on the Danish map in figure 1*c*). We observe that links with large membership to the *holiday* component connect smaller Danish islands (which are typical holiday destinations, see also electronic supplementary material, figure S5). Furthermore, such links have in general longer distance compared to links that are member of the *weekend* and *workday* components. In fact, the average geographical distance, weighted by membership, between connected pairs of municipalities in the three networks is 80.1 km±67 for the *holiday* component, 59 km±55 for the *weekend* component and 56.1 km±54 for the *workday* component.

### (b) Consistency across datasets

Having argued that mobility patterns between Danish municipalities can be decomposed using NMF into three interpretable components, it is natural to wonder if similar results apply to other datasets describing mobility within and across cities. In this section, we use Facebook data released by the Data-for-Good initiative [13] to study travel patterns in three European countries: France, Italy and Spain throughout 2020 (see Material and methods). We selected these three countries among those covered by the Facebook data, because they included periods preceding the first epidemic wave. The data describe the daily number of trips between spatial units at the second administrative level, where trips are computed across time windows of 8 h (see Material and methods). In Denmark, the second administrative subdivision corresponds to the 98 municipalities analysed in the previous section, in France to 101 ‘départements’, in Spain to 33 ‘comarcas’, and in Italy to 107 ‘province’. Note that, while both describing travel patterns, the Facebook data and the mobile phone data analysed in the previous section are constructed in different ways (see Material and methods), and *a priori* it is not trivial that the two dataset would capture the same aspects of travel behaviour. In particular, the Facebook data does not capture trips within areas. Entries of the mobility matrix such that the origin and destination areas are the same correspond to the number of individuals that did not leave the area where they reside across two consecutive time windows. This includes both individuals who travelled within their area of residence and individuals who did not leave their home location. Furthermore, the Facebook data has much more limited coverage compared to the Danish mobile phone data. The estimated user base is around 4.9% for France, 5.3% for Italy and 4.1% for Spain (see Material and methods for further elaboration). With these caveats, we now explore the Facebook data.

**Figure 2.**
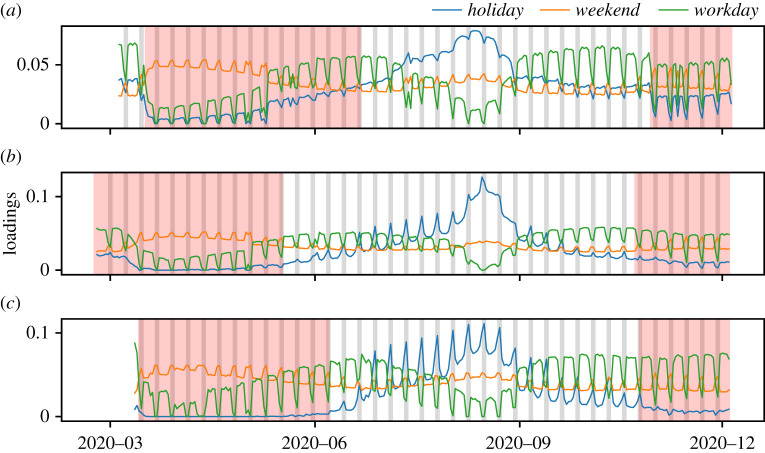
The components of mobility from Facebook data. The loadings ${\text{H}}_{k}$ of the three components of mobility identified by NMF over time in France (*a*), Italy (*b*) and Spain (*c*). The *holiday* component (blue, for k=1), the *weekend* component (orange, for k=2) and the *workday* component (green, for k=3). The red areas indicates the periods of ‘lockdown’ for each country. (Online version in colour.)

As above, we identify structural components in the mobility matrices describing movements in France, Spain and Italy by applying NMF. For consistency with the previous section, we set the number of components K=3. Then, we test the correlations between the identified components and the *holiday*, *weekend*, *workday*, and *covid-19 cases* vectors (table 2). Holidays include national holidays and summer vacations (see electronic supplementary material, table S1). For Italy and Spain, the results are remarkably similar to those obtained for Denmark, with the first component (k=1) correlating positively only with the *weekend* and *holiday* vectors; the second component (k=2) correlating positively only with the *weekend* and *covid-19 cases* vectors; and the third component (k=3) correlating positively only with the *workday* vector. In France, results are consistent with those obtained for the Spain and Italy with a small difference. The first component correlates positively with the *holiday* and *workday* vectors (see also figure 2*a*). This difference may be explained by the limited coverage of the French Facebook data. Importantly, results are consistent with those obtained in the previous section overall, implying that, across the two datasets, the three spatio-temporal components of mobility patterns identified by NMF have similar interpretations. As in the previous section, we name each component by the vector with highest correlation. We refer to the first component as the *holiday* component, the second as the *weekend* component and the third as the *workday* component.

**Table 2.** Pearson correlations between the three components found by NMF on mobility data collected by the Facebook Data-For-Good initiative (k∈{1,2,3}, see table rows), and the vectors describing *weekends*, *workdays*, *holidays* and *covid-19 cases* (see table columns). Results are shown for France, Italy and Spain. Unless stated in the table (ns), the correlations are significant at α=0.05.

		weekends	workdays	COVID-19 cases	holiday
France	k=1	−0.72	*0.72*	−0.62	*0.70*
	k=2	*0.86*	−0.86	0.38	0.04 (ns)
	k=3	−0.87	*0.87*	−0.19	−0.34
Italy	k=1	0.51	−0.51	−0.65	*0.72*
	k=2	*0.86*	−0.86	0.40	−0.21
	k=3	−0.91	*0.91*	−0.15	−0.08 (ns)
Spain	k=1	0.60	−0.60	−0.41	*0.79*
	k=2	*0.91*	−0.91	0.17	−0.05 (ns)
	k=3	−0.93	*0.93*	−0.06 (ns)	−0.15

We visualize the three components in the three countries in figure 2. Interestingly, through the decomposition into components, we observe that ‘lockdown’ periods have a similar effect on mobility patterns across countries. In particular, in all the studied countries, the periods of ‘lockdown’ results in a substantial decrease of commuting patterns, as captured by the *workday* component, but in an increase of leisure weekend patterns, as captured by the *weekend* component. On average, in comparison to other periods, the loading of the *workday* component during lockdown is 23% lower in Italy, 27% lower in France and 19% lower in Spain. Instead, the loading of the *weekend* components increased during periods of ‘lockdown’, by 25% in Italy, by 28% in France and by 17% in Spain.

### (c) The components of mobility and the effective distance between cities

How did the structure of the networks of flows capturing the different components of mobility change throughout the unfolding of the epidemics? In this section, focusing on the Danish data, we address this question by measuring the *effective distance* [29] (see Material and methods) between cities across the three components of mobility. The effective distance between two cities o and d is a quantity defined for networks representing flows of travellers between cities which measures how likely a traveller that leaves from node o will pass by node d. The effective distance is relevant to the study of epidemics spreading because it correlates with the arrival time of an emergent infectious disease [29]: it takes longer for a disease to spread to areas that are at a large effective distance from the origin of an outbreak compared to those that are at a short effective distance, irrespectively of their position in space.

We now define the effective distance. Take a directed network of cities, where links between any pair of cities are weighted based on the number of travellers between the two. For pairs of nodes i=(o,d) connected with a link, the effective distance is computed as $d_{i}=(1-\log P_{i})$, where $P_{i}$ is the fraction of travellers going to the destination d among all the travellers leaving the origin o. In general, the effective distance $D_{i}$ for an arbitrary pair of nodes i=(o,d), where the two are not necessarily connected, is defined as the length of the shortest path from o to d, where links along the path are weighted based on the effective distance defined above for pairs of connected nodes [29]. The effective distance does not correlate to the geographical distance between the two cities and is invariant to the amount of travel in a network.

Next, we assess how the *weekend*, *workday* and *holiday* components of mobility contribute to the effective distance between cities. In order to understand the contribution of each component k∈{weekend,workday,holiday}, and each day t, we consider a *partial network of flows*, that includes the trips of all components excluding k. The *partial networks of flows* are defined as follows. First, for each k, we compute the partial mobility matrix ${\text{P}}^{k}={\tilde{\text{W}}}_{k}{\tilde{\text{H}}}_{k}$, where ${\tilde{\text{W}}}_{k}$ is the membership matrix W without the column corresponding to component k, and ${\tilde{\text{H}}}_{k}$ is the matrix of loadings H without the row corresponding to component k. Just as the original mobility matrix A, ${\text{P}}^{k}$ has dimensions $N^{2}\times D$. Each entry $P_{i,t}^{k}$ of the matrix corresponds to the normalized number of trips between a pair of municipalities i=(o,d) on day t, excluding trips attributable to component k. For each day t, the partial mobility matrix ${\text{P}}^{k}$ naturally defines a network, where nodes are cities and weighted links correspond to all trips, excluding those related to a particular travel behaviour: either weekend, workday or holiday mobility. We use this procedure, instead of considering each mobility component individually, because that produces sparse networks. Finally, we compute the effective distance on the *partial mobility networks of flows* over time. Note that we have removed all links that, due to the way data is collected and anonymized, have median effective distance over time equal to zero (e.g. no travellers between them). This procedure resulted in the removal of 2592 out of 9604 links.

**Figure 3.**
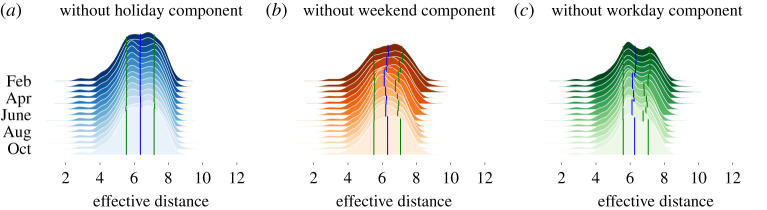
Effective distance over time in the partial networks of flows. Probability distributions of the effective distances over time in the three *partial networks of flows*. The partial networks are computed excluding the *holiday* (*a*), *weekend* (*b*) and *workday* (*c*) components, respectively. The blue lines correspond to the medians of each distribution, and the green lines to the 25th and 75th quantiles. Each distribution is computed considering all trips over a window of 20 days. (Online version in colour.)

With these definitions in place, we can now study the evolution of the effective distance in the partial mobility networks over the course of 2020 (figure 3). As expected, we observe that the mean effective distance across origin–destination pairs is approximately stable in time, because the effective distance is invariant to the amount of travel in a network. More interestingly, other properties of the distributions change over time. Let us first consider the variance of the distributions. Considering the *partial networks of flows* that include *weekend* and *workday* trips (figure 3*a*), the variance appears to be stable over time. This reveals that, overall, when considering all trips, the structure of the network remains relatively stable in time, despite the substantial reduction of trips. This finding is in line with previous results [11], showing that during periods of ‘lockdown’, the structure of the mobility network changes mostly due to the reduction of long-distance trips. Instead, when excluding *weekend* and *workday* trips (see figure 3*c*,*d* subplots, respectively), we observe that the variance of the distribution of effective distance was substantially larger during the ‘lockdown’ period (between March and May). The variance of the distribution changed from 1.19±0.02 during lockdown to 1.08±0.03 after lockdown when excluding *weekend* trips and from 1.22±0.005 during lockdown to 1.03±0.02 after lockdown when excluding *workday* trips. This finding reveals that, during periods of ‘lockdown’, there are more heterogeneity in the effective distance between cities, as it is more unlikely to travel to holiday destinations, and more likely to travel to close-by destinations. Overall, we observe that the lockdown has a substantial impact on the network structure only if holiday trips are included.

Let us then consider the shape of the distributions. Interestingly, by comparing the distributions across components, we observe substantial differences. We observe that, when including *holiday* and *weekend* trips, the distributions display a bimodal shape (figure 3*c*). A possible explanation for the observed bimodality lies in the fact that, when it comes to holiday and weekend travel, certain cities act as travel bridges, because they are located along major travel roads, and connect travellers from their home locations to their travel destinations, while other cities act as origins or destinations of the trip. In the former, the distribution of effective distances is extremely unbalanced, because trips occur mostly along one preferential travel axis. In the latter, the distribution of effective distances is more even. Instead, when including *holiday* and *workday* trips (figure 3*c*), the distributions are mostly unimodal. This may be due to the inherent structure of commuting trips, which dominate this network. In fact, commuting flows from a given origin are relatively evenly distributed, because the chance to commute at a given distance decreases with the distance [30]. Finally, the distributions including *holiday* and *workday* trips (figure 3*a*) are bimodal, even though the bimodality is less pronounced compared to the case when weekend trips are included. Importantly, we observe that the ‘lockdown’ does not affect the characteristic shapes of the distributions substantially. Finally, we note that the effective distance between pairs of cities, in the different networks considered, is partly explained by the geographical distance between them (see electronic supplementary material, figure S7).

## 3. Discussion

In this work, we investigated the effect of the restrictions imposed to contrast the spread of COVID-19 on mobility behaviour in four European countries. We used two mobility datasets describing daily trips between spatial units at the second administrative level throughout 2020, one collected by major Danish mobile phone operators and the other collected by Facebook in Spain, France and Italy. Using non-negative matrix factorization techniques, we argued that mobility patterns can be naturally decomposed into three travel components, describing weekend, workday and holiday mobility behaviour, respectively.

In line with previous results [2–9,11], we found that periods characterized by stringent restrictions, e.g. ‘lockdown’, resulted in a drastic reduction of overall travel. However, periods of ‘lockdown’ did not affect all components of travel equally. In particular, we found that, while the component capturing *workday* travel experienced a dramatic reduction during lockdown, the component describing *weekend* behaviour experienced an *increase* in the same periods. This result is consistent across all countries under study, suggesting that individuals’ travel behaviour during periods of lockdown was more similar to their behaviour during weekends in periods without restrictions. However, due to differences in data collection between the mobile phone and the Facebook datasets, the finding has to be interpreted in different ways across the countries under study. The data collected by the Danish mobile phone operators records all trips, including those within the same city. Thus, in Denmark, the data reliably describe travel behaviour, revealing that, during lockdown, people would travel mostly for leisure (e.g. walks, jogs and trips to weekend travel destinations). This observation is in line with the fact that, in Denmark, visits to parks increased during periods of lockdown according to mobility data made available by the Google Mobility Reports [31]. Denmark, in fact, experienced less stringent travel restrictions compared to the other European countries under study: ‘stay-at-home’ orders were never applied, and travel between different cities and regions remained always allowed [14]. Despite the surge in visits to parks and the increase of weekend-like mobility patterns during the first Danish lockdown, the containment strategies were effective. This results suggest that the correlations between mobility and social behaviour are non-trivial, implying that containment strategies aiming at reducing mobility do not necessarily target social behaviour.

Owing to the way it is pre-processed, the data released by Facebook does not account for within-city trips. Furthermore, the Facebook data does not allow to distinguish two different situations: individuals staying home versus individuals travelling within their home city. For this reason, the results obtained using the Facebook data may be partially explained by the fact that, during ‘lockdown’ individuals spent more time at home, just as they would do during weekends. In France, Italy and Spain the restrictions were in fact more stringent compared to Denmark, especially during the first epidemic wave. With ‘stay-at-home’ orders in place, people were only allowed to walk in the vicinity of their home location.

Our analysis shed light on how the identified travel components contribute to the structural property of the network describing travel flows between pairs of cities. In particular, we studied the *effective distance* [29], a network measure that quantifies the probability of travelling between two cities and correlates with the time it takes for a disease to travel between them. In line with previous findings [11], we found that, during periods of lockdown, the network describing flows of travellers experienced dramatic structural changes. We observed that, during periods of lockdown, there was larger heterogeneity in the effective distance between pairs of cities. Compared to periods without lockdown, it was much more likely to travel to specific cities, and much less likely to others. We found that this structural changes are explained, to a large extent, by what we identified as the *holiday* component. Thus, the decrease in holiday-related travel resulted in a more heterogeneous distribution of the effective distance between cities. This result is in line with the finding that changes in the structural properties of the network during lockdown are mostly due to the reduction in long distance trips [11]. Interestingly, it was shown that these structural changes have a considerable effect on epidemic spreading processes by delaying the spread to geographically distant regions [11]. Instead, we found that, despite the changes with respect to the amount of travel, the structure of the networks describing weekend and weekday mobility have remained substantially stable throughout the year.

Overall, our work shed light on how travel restrictions aiming at contrasting the spread of COVID-19 affected different aspects of travel behaviour, from commuting to going on vacation, and contributed structural changes of the travel flow network. Our work contributes to the stream of literature which focuses on the interplay between mobility behaviour and the spread of diseases. Understanding the effects of travel restrictions on behaviour and the spread of diseases is paramount to design effective policies for mitigating the ongoing COVID-19 pandemic and prepare for future epidemics.

## 4. Material and methods

### (a) Data description and pre-processing

#### (i) Danish telecommunication data

Analyses are based on aggregated mobility flows between municipalities estimated by mobile devices in the period between the 1 February 2020 and the 6 December 2020. The data are gathered from major mobile phone network operators. It consists of the number of daily trips between and within municipalities and has been released to help Statens Serum Institut (SSI) to model the spread of COVID-19 and understand population behaviour in response to various lockdown/mitigation measures. The dataset was officially requested by SSI and the legality of its use was ensured by the danish Ministry of Industry, Business and Financial Affairs. We have added a 5% noise to each data point to ensure the origin of the data is confidential. Data points consisting of fewer than 10 trips are removed to preserve anonymity. We further removed 9 days of data due to issues related with data release (see electronic supplementary material, figure S1). The data consist of a matrix with dimensions 9604×300, where 9604 is the number of pairs of cities, and 300 is the number of days considered.

#### (ii) Facebook data

We use so-called ‘Movement Maps’ at the second administrative level released by the Facebook’s Data for Good initiative [13]. Movement maps report the number of users that travel between areas, for three different 8 h time windows each day (00-8, 8-16, 16-00). Specifically, for each pair of areas i=(o,d), and each given time window t, the data reports the number of individuals that, during window t spent most of their time in d, and during the window t−1 preceding t spent most of their time in o. Note that the two areas o and d are not necessarily different, because individuals can spend most of their time in the same area in two consecutive windows (for more information see https://covid19.compute.dtu.dk/data-description/movement_maps/). We selected data for France, Spain and Italy among the countries covered by the Facebook Data-For-Good Initiative, because in these countries the data included periods preceding the first epidemic wave. We estimated that the data includes 4.9% of France’ population, 5.3% of Italy’s population and 4.1% of Spain’s population. These numbers are estimated by computing the number of individuals in each time window, then averaging across all time windows, then dividing by the population in each country.

#### (iii) Data pre-processing

We pre-processed the data as follows. First, for each day t, we normalized the mobility flows from a given origin o to a given destination d, dividing by the population of the origin city o. This transformation ensures that our description captures the number of trips per individual, and it is not strongly biased towards large cities. In fact, non-negative matrix factorization techniques are very sensitive to outliers. In line with previous literature [32,33], we transformed the data to reduce heterogeneity and limit the effect of outliers. By comparing the log-transformation and the square-root transformation (see electronic supplementary material, figure S2), we found the results of the two are comparable, but the latter yielded the most interpretable results. Thus, for any given day t, and any pair of cities i=(o,d), we pre-processed the data as follows:

4.1 $$A_{i,t}=\sqrt{\frac{f(o,d,t)}{pop(o)}},$$

where $A_{i,t}$ are entries of the final matrix used for the analysis, f(o,d,t) is the raw data describing the number of travellers between o and d on day t, and pop(o) is the population of o.

### (b) Non-negative matrix factorization

Given a data matrix $\text{X}\in R^{n\times m}$, the NMF is mathematically defined as

4.2 $$\min_{\text{W},\text{H}}F(\text{W},\text{H})=\min_{\text{W},\text{H}}\frac{1}{2}||\text{X}-\text{W}\text{H}|{|}_{F}^{2}\;\text{W}\in R_{+}^{n\times k}\;\text{H}\in R_{+}^{m\times k}\;k\in N,$$

where k is the hyper parameter specifying the inter-dimension between W and H. NMF was popularized by [19] who introduced a simple algorithm to solve equation (4.2). In this definition, the Frobenius norm is used as the distance measure between the matrices. By solving equation (4.2), the solution to the factorization of X is found to be

4.3 X≈WH.

The constraint of non-negativity assumes that the data, X, is non-negative. The hyper parameter k defines how many components one wants to find. NMF is a topic extraction tool that generates k sets of non-negative components that represent the weighted set of co-occurring features. To compute the NMF, we used the scikit-learn Python package, v. 0.23.2 [34] with random initialization and a maximum of 100 000 iterations. We used the ‘Coordinate descent’ solver [35]. We set the other parameters to the default values by scikit-learn.

#### (i) Selection of k

We selected the number of components k using the method proposed in [24]. The method estimates k by cross-validation. First, for each choice k, we computed the stability of the NMF solution using cross-validation. Then, we plotted the stability of the solution against the number of components k, and selected the best k using the elbow method (see electronic supplementary material, figure S3). The cross-validation routine for a matrix A consists of six steps.

(i) Define an holdout set of size n by setting a row holdout set $I_{l}\subset \{1,\ldots ,n\}$, and a column holdout set $I_{j}\subset \{1,\ldots ,n\}$.
(ii) Find the NMF solution $(\tilde{\text{W}},\tilde{\text{H}})=\arg\min_{\text{W},\text{H}\geq 0}\sum_{t}||{\text{A}}_{-I_{l},-I_{j}}-\text{W}\text{H}|{|}_{F}^{2}$, where ${\text{A}}_{-I_{l},-I_{j}}$ is the original matrix without the rows and columns forming the holdout set.
(iii) Find the NMF solution $\overset{ˇ}{\text{W}}=\arg\min_{\text{W}\geq 0}\sum_{t}||{\text{A}}_{I_{l},-I_{j}}-\text{W}\tilde{\text{H}}|{|}_{F}^{2}$, where ${\text{A}}_{I_{l},-I_{j}}$ is the original matrix without the columns in the column holdout set.
(iv) Set $\overset{ˇ}{\text{H}}=\arg\min_{\text{H}\geq 0}\sum_{t}||{\text{A}}_{-I_{l},I_{j}}-\tilde{\text{W}}\text{H}|{|}_{F}^{2}$, where ${\text{A}}_{-I_{l},I_{j}}$ is the original matrix without the rows in the row holdout set.
(v) Set $\hat{\text{A}}=\overset{ˇ}{\text{W}}\overset{ˇ}{\text{H}}$
(vi) Compute the test error: $\text{Test Error}=\sum_{t}||\text{A}-\hat{\text{A}}|{|}_{F}^{2}$

We computed the average test error, across choices of k, by cross-validation. We set the size of the test set n=40. Finally, we plotted the test error against k. Using the ‘elbow method’ [36] (see electronic supplementary material, figure S3), we found the best choice of k to be k=3.

### (c) Measures we use to quantify the differences in the networks

#### (i) Effective distance

The effective distance is a network metric introduced in [29]. Given a network of flows between origin–destination pairs, and a given origin–destination pair i=(o,d), where o and d are connected via a link, the effective distance is defined as

4.4 $$d_{i}=(1-\log P_{i})\leq 1,$$

where $P_{i}$ is the fraction of trips going from o to d relative to all trips leaving node o. Note that the measure is generally asymmetric as $P_{i=(o,d)}\neq P_{i=(d,o)}$, reflecting the asymmetry of real world travel. Note also that $d_{i}$ is dimensionless and unaffected by the magnitude of the mobility flow.

More in general, to account for the fact that not all pairs of cities are connected via a link, the authors of [29] defined the effective distance between any pairs of network nodes as follows. Assume $\Gamma =\{n_{1},n_{2},\ldots ,n_{L}\}$ is an ordered path from any two nodes $n_{1}$ to $n_{L}$ and $W(\Gamma )=\prod_{i-1}^{L-1}P_{n_{i+1}n_{i}}$, then the distance of that path is defined as

4.5 λ(Γ)=L−logW(Γ).

The effective distance, $D_{i=(o,d)}$, is then the path that minimizes the distance

4.6 $$D_{nm}=\min_{\Gamma }\lambda (\Gamma ),$$

which is equivalent to computing the shortest path in a network with weights computed from the effective distance (equation (4.4)) plus one.

### (d) Cartograms

We display the maps of Denmark in figure 1 as cartograms [17]. We computed the cartograms as follows. First, we considered the spatial polygons describing the area covered by each Danish municipality. Then, we altered the size of each polygon to ensure that the area covered by each polygon in the map is proportional to the population of the corresponding municipality. We used the iterative flow-based algorithm developed by [17] to compute the cartograms and ensure that the structural connections between polygons is preserved.

## Data accessibility

The Facebook data to reproduce the findings of this paper can be found at https://github.com/Udneowi/Covid19_mobility_NMF. Data from the Danish mobile phone operators cannot be shared due to the agreement with the companies. All code necessary to reproduce results is available at: https://github.com/Udneowi/Covid19_mobility_NMF.
